# Twenty Years in a Trance

**Published:** 1886-11

**Authors:** 


### TWENTY YEARS IN A TRANCE.

### MISS MOLLIE FANCHER STILL ALIVE AND SLOWLY RECOVERING HER VITALITY.

In a three-story brown-stone front in Brooklyn lives Miss Mollie Fancher. It was she who twelve years ago attracted universal attention as a mind-reader. Her history is out of the ordinary and has a tinge of sadness in it. Over twenty years ago she was one of the most promising and bright pupils of Prof. West's Academy in Montague street. She was then young, pretty and vivacious. There was nothing, however, in her appearance that would indicate that she possessed such wonderful powers as were afterwards developed through several accidents. She at that time did not know that she possessed such extraordinary gifts. One day when she was out horseback riding, the animal became frightened and she was thrown to the ground, seriously injured. She recovered from the effects of the fall only to meet with a more serious accident which crippled her for life. She was stepping from a horse-car when her dress became entangled in the step. The car started and she was dragged over half a block. She was taken to her home unconscious. The accident brought on nervous prostration and other diseases which caused her intense agony for many months. For several days she was in a trance and had all the appearance of a corpse. The doctors believed her to be lifeless. She finally recovered, but an awful change had taken place. The beautiful young girl was transformed to a distorted and crippled woman. She was blind and her lower limbs were twisted out of shape. Her hands were held at the back of her head and her arms could be moved only with great difficulty. It was then that she displayed her remarkable power of mind-reading. A certain physician had a valuable set of surgical instruments stolen from him. While pondering over the robbery he called on Miss Fancher. As he came in her room she said: "Doctor, I am really sorry for your loss, but trust that you will soon recover the instruments." He was taken by surprise at Miss Fancher's divining his thoughts. No one had spoken to her about the robbery.

Miss Fancher was offered a fortune by Barnum to display her powers in public. She refused. She is now a middle-aged, pleasant lady, living with her aunt, Miss Crosby. She has recovered the use of her hands and retains her power of mind-reading, and stated the other day that although her eyes are closed she can see as well as most people in possession of their sight. Her history would fill volumes and would be of great interest, but many Brooklynites are still familiar with the accounts of her powers that appeared in the papers a number of years ago.—*N Y. World.*

